# Evaluating ChatGPT’s moral competence in health care-related ethical problems

**DOI:** 10.1093/jamiaopen/ooae065

**Published:** 2024-07-09

**Authors:** Ahmed A Rashid, Ryan A Skelly, Carlos A Valdes, Pruthvi P Patel, Lauren B Solberg, Christopher R Giordano, François Modave

**Affiliations:** Department of Anesthesiology, University of Florida College of Medicine, Gainesville, FL 32608, United States; Department of Anesthesiology, University of Florida College of Medicine, Gainesville, FL 32608, United States; Department of Surgery, University of Florida College of Medicine, Gainesville, FL 32608, United States; Department of Research, Alabama College of Osteopathic Medicine, Dothan, AL 36303, United States; Department of Community Health and Family Medicine, University of Florida College of Medicine, Gainesville, FL 32608, United States; Department of Anesthesiology, University of Florida College of Medicine, Gainesville, FL 32608, United States; Department of Anesthesiology, University of Florida College of Medicine, Gainesville, FL 32608, United States

**Keywords:** artificial intelligence, clinical decision-making, ethics, judgement, morals

## Abstract

**Objectives:**

Artificial intelligence tools such as Chat Generative Pre-trained Transformer (ChatGPT) have been used for many health care-related applications; however, there is a lack of research on their capabilities for evaluating morally and/or ethically complex medical decisions. The objective of this study was to assess the moral competence of ChatGPT.

**Materials and methods:**

This cross-sectional study was performed between May 2023 and July 2023 using scenarios from the Moral Competence Test (MCT). Numerical responses were collected from ChatGPT 3.5 and 4.0 to assess individual and overall stage scores, including C-index and overall moral stage preference. Descriptive analysis and 2-sided Student’s *t*-test were used for all continuous data.

**Results:**

A total of 100 iterations of the MCT were performed and moral preference was found to be higher in the latter Kohlberg-derived arguments. ChatGPT 4.0 was found to have a higher overall moral stage preference (2.325 versus 1.755) when compared to ChatGPT 3.5. ChatGPT 4.0 was also found to have a statistically higher C-index score in comparison to ChatGPT 3.5 (29.03 ± 11.10 versus 19.32 ± 10.95, *P =.*0000275).

**Discussion:**

ChatGPT 3.5 and 4.0 trended towards higher moral preference for the latter stages of Kohlberg’s theory for both dilemmas with C-indices suggesting medium moral competence. However, both models showed moderate variation in C-index scores indicating inconsistency and further training is recommended.

**Conclusion:**

ChatGPT demonstrates medium moral competence and can evaluate arguments based on Kohlberg’s theory of moral development. These findings suggest that future revisions of ChatGPT and other large language models could assist physicians in the decision-making process when encountering complex ethical scenarios.

## Introduction

Ethics is the systematic study of principles of moral “correctness” that guide decision-making when facing moral questions.^1^ While the training of physicians is vigorous with various multidimensional components, there is a strong demand for further teaching in ethics to develop competence in assessing ethical problems.^2^ The process of making ethically complex decisions can be influenced by a physician’s moral development and this morality is shaped by individual experiences, such as upbringing, religion, and socioeconomic factors.^3–5^ Moral development, as theorized by Dr. Lawrence Kohlberg, consists of 6 stages of development that are grouped into 3 levels: preconventional (*Obedience and Punishment* and *Self-Interest*), conventional (*Interpersonal Relationships* and *Law and Order*), and postconventional (*Social Contract* and *Universal Principles*), each of which provides a depth of understanding and a different approach to moral questions (Table 1).^6^

**Table 1.** Lawrence Kohlberg’s stages of moral development.

Level and age	Stage	Description
Preconventional up to 9 years of age	Obedience and punishment	Moral reasoning is based on direct consequences of their actions.
	Self-interest	Moral reasoning is driven by self-interest and rewards.
Conventional Early adolescence to adulthood	Interpersonal accord and conformity	Governed by interpersonal relationships and conformation to societal norms. Effort is made to remain in good social standing with others.
	Law and order	Greater importance is given to upholding societal laws and maintaining social order.
Postconventional Some adolescent and adults (20%-25% of adult population)	Social contract	Individuals interpret rules as social agreements rather than absolute laws or mandates. Moral and legal correctness are not always the same.
	Universal principles	Individuals develop internal moral principles that are upheld above societal law.

Recent advancements in artificial intelligence (AI) have catalyzed transformative movements across various industries, including medicine. The integration of AI in medicine has the potential to facilitate the delivery of health care across specialties.^7–9^ Traditionally, medicine has relied on human knowledge and experience, and while effective this approach is limited by subjectivity, bias, and time. AI programs, however, have the capability of learning from very large datasets, recognizing complex patterns, and providing recommendations based on available evidence, thus minimizing these shortcomings. While the use of AI has led to advancements in imaging, differential diagnosis, and treatments, there is scarce literature on the use of AI for health care-related ethical problems.^10–12^

Chat Generative Pre-trained Transformer (ChatGPT; OpenAI, San Francisco, CA, United States) is a large language model-based (LLM) chatbot, released in 2021, that was developed using reinforcement learning with human feedback technique to generate conversations with a user.^13^ When presented with a prompt, ChatGPT will generate a response based on thousands of online sources collected from the internet. In the past, ChatGPT has been used in various health care and non-health care-related applications.^14–16^ However, ChatGPT’s ability to evaluate ethically complex medical decisions has not been thoroughly studied.^17^^,^^18^ The goal of this study is to assess the reliability of ChatGPT’s moral competence when faced with ethically challenging scenarios. Next, we seek to compare the advancement in ChatGPT’s performance when looking at different iterations of ChatGPT, specifically 3.5 versus 4.0. Lastly, we will quantify the current level of ChatGPT’s moral competence.

Understanding ChatGPT’s capabilities and limitations in this context is essential for the future integration of AI into clinical practice. Our study provides an initial assessment of ChatGPT’s moral competence, which will assist future researchers in developing AI tools that are not only proficient but also enhanced decision making in the clinical setting.^19^

## Methods

As this study focuses on various moral concepts, it is worth noting the differences between the various terminology used in the literature. Moral reasoning is defined as the cognitive process that people use to differentiate right and wrong in situations and how they acquired such principles.^20^ Moral development refers to age-related stages that people experience when evaluating morality and is often used as the principles in moral reasoning for decisions such as Kohlberg’s stages of moral development.^21^ The ability to consistently deliberate and interpret the decisions for ethical problems based on Kohlberg’s stages of moral development is moral competence.^22^

To assess ChatGPT’s moral competence, Dr. Georg Lind’s Moral Competence Test (MCT), previously known as Moral Judgement Test, was used. Dr. Lind used Kohlberg’s stages of moral development to create a test that isolated and objectively measured individual morality separately from opinions and beliefs.^22^^,^^23^ The MCT provides quantitative analysis on moral assessment with prior use in health care research, making it an ideal tool for evaluating LLM such as ChatGPT.^3^^,^^4^^,^^24^^,^^25^ This test consists of 2 ethical scenarios: a health care-based “doctor’s dilemma” (a physician is faced with a life-saving dilemma) and a non-health care-based “workers’ dilemma” (2 workers are confronted with a dilemma related to illegal work behavior).^6^^,^^23^ Each scenario had 12 statements, all of which corresponded to 1 of the 6 stages of Kohlberg’s theory of moral development: 6 supporting the proposed action (pro argument) and 6 opposing it (con argument). ChatGPT was presented with both scenarios and each of the corresponding statements. After each statement, ChatGPT was asked to respond on a 9-point Likert-type scale, from −4 (completely unacceptable) to +4 (completely acceptable), for each statement.^23^^,^^26^

The C-index, a summarized score on a scale of 1-100, was subsequently calculated to analyze all numerical responses collected from both scenarios. Per the MCT, this metric gauges an individual’s capacity to evaluate an argument through the lens of their moral interpretation while considering the extent to which personal judgments are influenced by moral considerations or principles, as opposed to personal opinions and constructs.^23^^,^^26^ A high C-index score indicates that an individual’s moral judgments are consistent and based on moral principles, regardless of whether these judgments coincide with their own opinions. This implies a higher level of moral competence. Conversely, a low C-index score suggests that a person allows their view on the “right” solution of a dilemma determine the ratings of counter arguments. The C-index does not measure an individual’s opinions or judgements to a dilemma but rather the consistency at which they apply their moral principles. C-index scores below 9 are considered to be *low*, scores 10-29 are considered *medium*, scores above 30 are considered *high* (Table 2).^23^^,^^26^ All C-indices were calculated using in-house Python software (version 3.10.9). Along with C-index, the MCT provides scores for each of the 6 stages of moral development built into the test, referred to as moral preference or attitude.^27^ Each stage can then be averaged of the 4 arguments involved (2 doctors, 2 workers), creating an overall preference ranging from −4 (completely unacceptable) to +4 (completely acceptable). All averages of the 6 stages of moral development were also calculated using in-house Python software (version 3.10.9).

**Table 2.** C-index assessment.^22^

C-index range	Level of moral competence
1-9	Low
10-29	Medium
>30	High

Data were collected from May 2023 to July 2023 and included the use of online edition ChatGPT 3.5 (model=text-davinci-002-render-sha) and ChatGPT 4.0 (model=gpt-4). All inputs were standardized by a committee of reviewers (A.R., R.S., P.P.) and each prompt was submitted individually on ChatGPT. Both models were performed in isolation, simultaneously with separate memberships. For analysis, each iteration of the MCT performed by ChatGPT 3.5 and 4.0 was treated as an individual response of the affiliated group. A total of 100 complete iterations were compiled and split equally. Minor grammatical revisions for certain prompts were implemented accordingly and reviewed by a committee (A.R., R.S., P.P.). Prompts and responses were collected by in-house Python software (version 3.10.9) and further parsed into organized datasets for preliminary data validation. Each iteration was validated by a 2-stage process, where 2 independent reviewers (R.S., P.P.) validated the dataset initially and a third reviewer (A.R.) performed an independent validation assessment. Incomplete responses that did not provide both qualitative and quantitative data responses were excluded from data analysis and regenerated.

Descriptive analysis, including mean and standard deviation, was performed for all continuous data variables in each scenario of the MCT. Two-sided Student’s *t*-test was used to assess significant differences between continuous variables in comparison from ChatGPT 3.5 and ChatGPT 4.0. A *P* value of <.05 was considered statistically significant.

## Results

**Figure 1.**
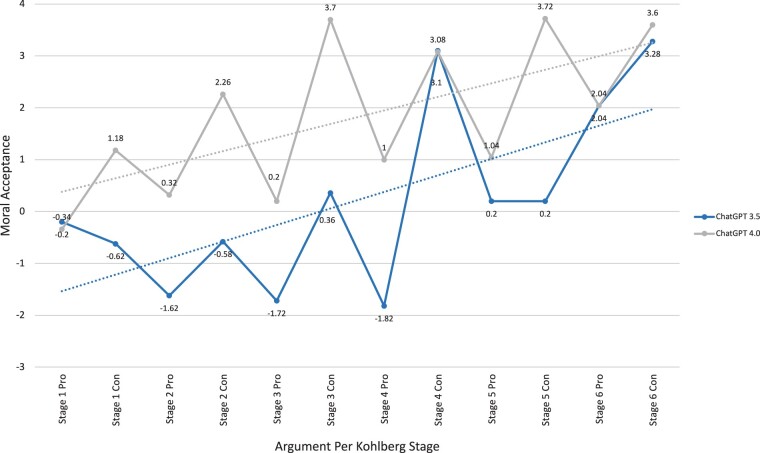
Moral preference of workers’ dilemma.

A total of 100 complete iterations of the MCT were performed and equally distributed to ChatGPT 3.5 and ChatGPT 4.0. For the workers’ dilemma, the lowest scores of moral preference were noted for the Pro Stage 4 for ChatGPT 3.5 (−1.82 ± 0.96) and the Pro Stage 1 for ChatGPT 4.0 (−0.34 ± 1.17). The highest scores of moral preference were noted for the Con Stage 6 for ChatGPT 3.5 (3.28 ± 1.03) and the Con Stage 5 for ChatGPT 4.0 (3.72 ± 0.45) (Table 3). Moral preference was found to be generally higher for the Con scenarios in both ChatGPT 3.5 and 4.0 models as compared to the Pro scenarios indicating that both models disfavored the workers’ action against the company (Figure 1). Comparatively, ChatGPT 3.5 and ChatGPT 4.0 differed significantly when evaluating moral preference in all but 4 scenarios (Pro Stage 1, Con Stage 4, Pro Stage 6, Con Stage 6) with higher moral preference generally in the later stages (*P* = 1.49*10^−2^ – 1.46*10^−19^).

**Table 3.** Comparison of ChatGPT 3.5 and 4.0 per Kohlberg stage for workers’ and doctor’s dilemmas.

Workers’ dilemma						
		ChatGPT 3.5		ChatGPT 4.0		
		Mean score	Standard deviation	Mean score	Standard deviation	*P* value
Stage 1	Pro	−0.2	1.98	−0.34	1.17	6.69E-01
	Con	−0.62	1.51	1.18	1.08	9.28E-10^a^
Stage 2	Pro	−1.62	1.22	0.32	0.99	1.22E-13^a^
	Con	−0.58	1.69	2.26	0.52	2.19E-16^a^
Stage 3	Pro	−1.72	0.83	0.2	0.85	1.46E-19^a^
	Con	0.36	2.81	3.7	0.46	4.73E-11^a^
Stage 4	Pro	−1.82	0.96	1	0.8	1.78E-28^a^
	Con	3.1	0.99	3.08	0.34	8.93E-01
Stage 5	Pro	0.2	2.2	1.04	0.87	1.49E-02^a^
	Con	0.2	2.93	3.72	0.45	3.51E-11^a^
Stage 6	Pro	2.04	1.6	2.04	0.78	1.00E + 00
	Con	3.28	1.03	3.6	0.49	5.18E-02

Doctor’s dilemma						
		ChatGPT 3.5		ChatGPT 4.0		
		Mean score	Standard deviation	Mean score	Standard deviation	*P* value
Stage 1	Pro	1.5	2.06	−2.06	0.79	5.86E-17^a^
	Con	−1	1.22	1.4	1.03	9.32E-18^a^
Stage 2	Pro	1.04	1.98	−2.66	1.13	2.60E-18^a^
	Con	−1.82	1.52	−2.16	1.03	1.95E-01
Stage 3	Pro	0.66	1.5	−0.98	0.97	6.62E-09^a^
	Con	−0.62	1.7	−0.02	1.28	4.96E-02^a^
Stage 4	Pro	2.56	0.86	−0.1	1.31	6.59E-20^a^
	Con	−1.02	1.84	2.86	0.9	4.61E-21^a^
Stage 5	Pro	3.42	0.78	2.72	1.17	7.54E-04^a^
	Con	−1.62	1.82	0.26	1.99	3.70E-06^a^
Stage 6	Pro	2.78	1.01	2.2	1.26	1.30E-02^a^
	Con	−1.08	1.97	1.46	1.9	2.88E-09^a^

a *P <*.05.

**Figure 2.**
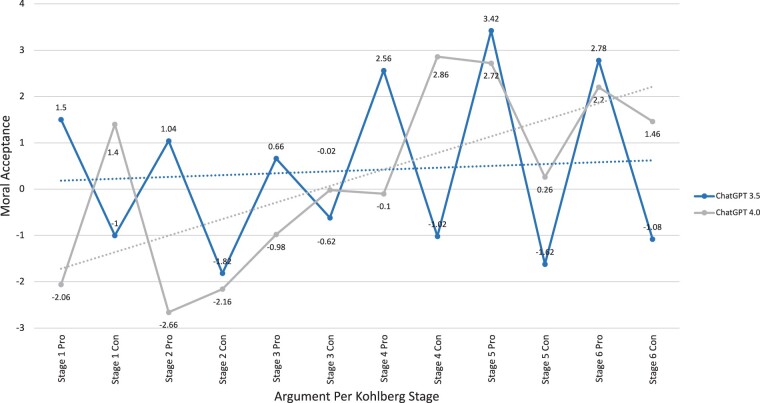
Moral preference of doctor’s dilemma.

For the doctor’s dilemma, the lowest scores of moral preferences were noted for the Con Stage 2 for ChatGPT 3.5 (−1.82 ± 1.52) and Pro Stage 2 for ChatGPT 4.0 (−2.66 ± 1.13). The highest scores of moral preference were noted for the Pro Stage 6 for ChatGPT 3.5 (2.78 ± 1.01) and the Con Stage 4 for ChatGPT 4.0 (2.86 ± 0.9) (Table 3). Moral preference was found to be generally higher for the Pro scenarios in both ChatGPT 3.5 and 4.0 models as compared to the Con scenarios indicating that both models favored the doctor’s action of performing euthanasia (Figure 2). Similarly to the workers’ dilemma, when comparing ChatGPT 3.5 to ChatGPT 4.0 moral preference differed significantly in all but 1 scenario (Con Stage 2) with higher moral preference generally in the later stages (*P* = 1.3*10^−2^ – 6.59*10^−20^).

**Figure 3.**
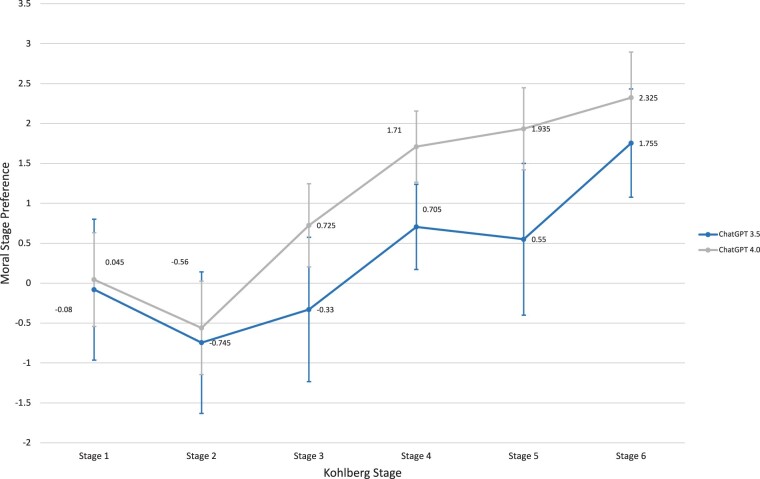
Overall preference of ChatGPT 3.5 versus 4.0.

When assessing overall moral stage preference for the workers’ and doctor’s dilemma combined, both models showed an upward trend towards the later stages including *Social Contract* (*Stage 5*) and *Universal Principles* (*Stage 6*). The highest moral stage preference was *Universal Principles* (*Stage 6*) with ratings of 1.755 and 2.325 for ChatGPT 3.5 and 4.0, respectively. Both models also showed a characteristic dip on *Self-Interest* (*Stage 2*) which had the lowest score overall: −0.745 and −0.56 for ChatGPT 3.5 and 4.0, respectively. ChatGPT 4.0 also provided higher moral stage preference at later stages compared to ChatGPT 3.5. Of all the Kohlberg’s stages, *Law and Order* (*Stage 4*) was found to have the smallest standard deviation in moral stage preference for ChatGPT 3.5 (±0.53) and 4.0 (±0.44) (Figure 3).

In terms of moral competence, ChatGPT 3.5’s average C-index was found to be 19.32 ± 10.95 and classified as medium moral competence per Lind’s assessment. Interestingly, ChatGPT 4.0’s average C-index was 29.03 ± 11.10 and classified as medium. The higher C-index derived from ChatGPT 4.0 was found to be statistically significant (*P =.*0000275) (Table 4).

**Table 4.** Independent samples *t*-test for moral competency C-index between ChatGPT 3.5 and 4.0.

Group	*N*	Mean	Standard deviation	*t*	*P* value
ChatGPT 3.5	50	19.32	10.95		
				−4.40	2.75E-05^a^
ChatGPT 4.0	50	29.03	11.10		

a *P <*.05.

## Discussion

Existing literature has reported various health care-related applications of ChatGPT.^14–16^ However, this is the first quantitative study to evaluate ChatGPT’s ability to assess ethically complex medical arguments via MCT. Based on the results of our study, we believe that although the use of AI in the field of medical ethics is in its nascent stages, AI has the potential to be a useful tool in the clinical environment.

In our study, ChatGPT 3.5 and 4.0 trended towards higher moral preference for the later stages of Kohlberg’s theory for all dichotomous arguments in the workers’ and doctor’s dilemmas (Figures 1 and 2). This indicates that ChatGPT has the ability to discern components of an argument rather than opinion. Comparatively, however, ChatGPT was less successful at discriminating between the moral preference of Kohlberg-derived arguments in the doctor's dilemma than the workers’ dilemma. ChatGPT’s decreased ability to evaluate health care-related ethical scenarios limits its practical implication in the field of medicine and highlights the need for further AI development. Nevertheless, results from both dilemmas individually suggest that ChatGPT 4.0 is superior to ChatGPT 3.5 at evaluating moral preference, and further models should continue to support this concept. The summative moral stage preference shows similar findings with the newer rendition of ChatGPT exhibiting an increased ability to evaluate moral arguments (Figure 3).

While some Kohlberg stages had higher overall preference than others, *Law and Order* (*Stage 4*) was found to have the smallest standard deviation in both models indicating the highest consistent evaluation of all the stages. Often, society views laws as a safety net to provide social order, due to its dichotomous nature—legal and illegal. This same characteristic mimics binary classification, a common AI task, that explains ChatGPT’s consistency when assessing arguments related to law. Ideally, future models of ChatGPT should have similar consistency for all arguments, binary and nonbinary, for them to be useful tools for physicians.

ChatGPT showed the greatest moral preference for opposing arguments of the workers’ dilemma and, to a lesser extent, the supporting arguments in the doctor’s dilemma (Figures 1 and 2). This natural predisposition of ChatGPT’s evaluation suggests that despite its ability to discern moral arguments logically, ChatGPT has an internal moral opinion. While the MCT limits its assessment scores based on an undifferentiated moral competence rather than opinion, the test does create ambiguity/impartialness, thus hindering its application to real life scenarios that require definitive action. Given the state of AI and the nature of the MCT, it is difficult to assess ChatGPT’s moral compass and further studies are required.

Existing literature has questioned ChatGPT’s use as a moral judge.^17^^,^^28^ For example, Krügel and colleagues found that ChatGPT lacked a firm moral stance when given ethical prompts to advise subjects.^17^ Using the MCT, we found that ChatGPT exhibited medium to high moral competence with ChatGPT 4.0 having a significantly higher C-index compared to ChatGPT 3.5. With the global desire for AI to undergo accelerated improvement and become functionally useful, our findings suggest ChatGPT’s current development is fruitful and has potential to aid in real-world health care scenarios. The C-index we derived from ChatGPT is comparable to the moral competence of various physicians in training.^24^^,^^25^ However, our results also showed large variation in C-index, with some iterations falling in the “low” category. These variations limit ChatGPT’s current use in real-world ethical scenarios due to the potential for inconsistency and thus inequity, as well as possible impact on patient safety. Thus, we advise in-house training of prior ethical problems and AI-related communication workshops for health care workers to support the upcoming reality of utilizing AI models in medicine.

Overall, we recognize that our study does not indicate the current use of LLM like ChatGPT within the clinical setting in its current state. Ethical dilemmas are complex scenarios requiring deep cognitive processes that require multiple experienced individuals to come to a consensus. However, we do believe that with advancement in LLM there is potential to have AI tools to help physicians with making a decision in an ethical scenario.

Some additional potential applications for LLM in medical ethics are the use of it as an ethical educational tool and to help aid in further development of ethical AI tool. First, ChatGPT is capable of providing varying scenarios to users due to its ability to process large amounts of data and build algorithms based on patterns and probabilities. Medical students can generate case studies by using ChatGPT or other LLMs to provide them with a set of challenging ethical scenarios—allocation of resources, maternal-fetal conflicts, informed consent—to enhance their competence in ethical problems. The use of ChatGPT in medical education is limited and further applications beyond medical ethics should be explored.^2^^,^^16^^,^^29^

Our study has limitations. The first limitation to the study is the MCT itself. This is a test that evaluates moral competence but does not determine the stage of moral development someone exhibits based on Kohlberg’s theory of moral development. Therefore, although we believe our results are important to future applications of AI in medicine, we cannot definitively cite ChatGPT’s stage of moral development. Furthermore, Kohlberg’s theories of moral development have also been called into question since its initial focus was on childhood development, using only male participants, and an educational objective.^21^^,^^30^^,^^31^ A second limitation is the MCT was initially meant to be used to compare groups not individuals. Despite both models of ChatGPT having a degree of variance and tested in isolation mimicking a cohort-like structure, the MCT was not originally intended for serial testing of the same “participant” with no intervention performed. A third limitation is the restricted access to run ChatGPT 4.0 queries. Thus, we limited the number of data inputs to 50 each for ChatGPT 3.5 and 4.0. Future studies should evaluate AI using multiple data inputs to increase statistical power. Further, our study included only one health care dilemma. Future studies should evaluate moral competence using multiple health care dilemmas based on a variety of topics. While both versions of ChatGPT showed novel responses to the MCT from a scoring standpoint and decision-making process, we are unaware if the 2 dilemmas presented were part of the original training data used to train ChatGPT. An overtrained LLM could limit its feasibility for clinical use and can be avoided by having various unique healthcare dilemmas in both the train and testing data. Lastly, the prompts from the MCT did not provide local context. Thus, some responses given suggested referring to state laws, instead of answers based on morality. Future studies should provide context for ethical problems and compare the responses of multiple AI chatbots.

## Conclusion

Although the use of AI in the field of medical ethics is in its nascent stages, AI has the potential to be a useful tool in the clinical environment. Our study suggests that ChatGPT demonstrates medium moral competence, as assessed by Lind’s MCT, and can be used to evaluate arguments based on Kohlberg’s theory of moral development. Although increased variability in C-index and other markers were noted, we believe future developments and improvements of ChatGPT and other related LLM AI models have the potential to assist physicians in the decision-making process when encountering a difficult ethical decision.

## Data Availability

The data used in this study is freely available for any purpose, without investigator support.
